# Tool Compounds Robustly Increase Turnover of an Artificial Substrate by Glucocerebrosidase in Human Brain Lysates

**DOI:** 10.1371/journal.pone.0119141

**Published:** 2015-03-12

**Authors:** Zdenek Berger, Sarah Perkins, Claude Ambroise, Christine Oborski, Matthew Calabrese, Stephen Noell, David Riddell, Warren D. Hirst

**Affiliations:** 1 Pfizer Neuroscience Research Unit, 610 Main Street, Cambridge, MA, United States of America; 2 Pfizer Structural Biology Group, Eastern Point Road, Groton, CT, United States of America; 3 Pfizer Primary Pharmacology Group, Eastern Point Road, Groton, CT, United States of America; Emory University, UNITED STATES

## Abstract

Mutations in glucocerebrosidase (GBA1) cause Gaucher disease and also represent a common risk factor for Parkinson’s disease and Dementia with Lewy bodies. Recently, new tool molecules were described which can increase turnover of an artificial substrate 4MUG when incubated with mutant N370S GBA1 from human spleen. Here we show that these compounds exert a similar effect on the wild-type enzyme in a cell-free system. In addition, these tool compounds robustly increase turnover of 4MUG by GBA1 derived from human cortex, despite substantially lower glycosylation of GBA1 in human brain, suggesting that the degree of glycosylation is not important for compound binding. Surprisingly, these tool compounds failed to robustly alter GBA1 turnover of 4MUG in the mouse brain homogenate. Our data raise the possibility that *in vivo* models with humanized glucocerebrosidase may be needed for efficacy assessments of such small molecules.

## Introduction

Glucocerebrosidase is a lysosomal protein which breaks down glucosylceramide into glucose and ceramide [1,2]. Two mutant alleles in the glucocerebrosidase gene cause a lysosomal storage disorder called Gaucher disease. The mutations in glucocerebrosidase often cause decreased protein stability or enzymatic activity [2–5]. In Gaucher patients, glucocerebrosidase activity is decreased to ~ 5–20% of normal levels [3] and is accompanied by increased levels of its natural substrate, glucosylceramide [6]. The current treatment is peripheral administration of the active enzyme, glucocerebrosidase [5].

GBA1 dysfunction has also been recently linked to Parkinson’s disease (PD) and Dementia with Lewy bodies (DLB). Mutations in GBA1 represent a common risk factor for both diseases [7,8]. In addition, decreased GBA activity has been also observed in brain lysates from patients. Approximately 50–70% of normal activity was reported in brain lysates from PD patients [9] and ~ 75–80% of normal activity was seen in brain lysates from DLB patients [10]. Similar effects of decreased GBA1 activity and protein levels in PD brains were observed in an independent study [11]. These data collectively suggest that increasing GBA1 activity via a small molecule may be a viable therapeutic strategy for Gaucher and Parkinson’s diseases as well as for Dementia with Lewy bodies. Such an approach may be particularly attractive for neuropathic forms of Gaucher disease, PD and DLB due to a lack of disease modifying therapies (the peripherally administered protein does not cross the blood brain barrier).

Despite considerable screening efforts, GBA1 has proved to be a difficult target, yielding only small molecule GBA1 inhibitors [12,13]. Although these molecules can also act as chaperones leading to higher levels of GBA1 protein [14–17], the net effect on GBA1 activity will be increased GBA1 levels minus the degree of GBA1 inhibition. This may potentially explain the disappointing results in the phase 2 trial of Gaucher disease, where only one out of eighteen patients exhibited “clinically meaningful improvements in key measures of disease” [18].

Recently, novel non-inhibitory small molecules targeting GBA1 were reported [19]. These tool compounds increased activity of mutant N370S GBA1, measured by increased turnover of an artificial substrate 4MUG (4-Methylumbelliferyl β-D-galactopyranoside) in human spleen lysates [19]. In the present study, we set out to investigate the effect of two of these compounds (compounds 40 and 43) on GBA1 in brain lysates. We first show that these tool compounds can increase 4MUG turnover by wild-type human GBA1 protein in a cell free system. In addition, we demonstrate that they exert a robust effect on human brain-derived GBA1. Surprisingly, these tool compounds failed to robustly modulate GBA1 in mouse brain lysates. Since purified human GBA1 was able to increase 4MUG turnover when added to mouse lysates, our findings raise the possibility that *in vivo* models with humanized glucocerebrosidase may be needed for efficacy assessments of such small molecules.

## Results

**Fig 1:**
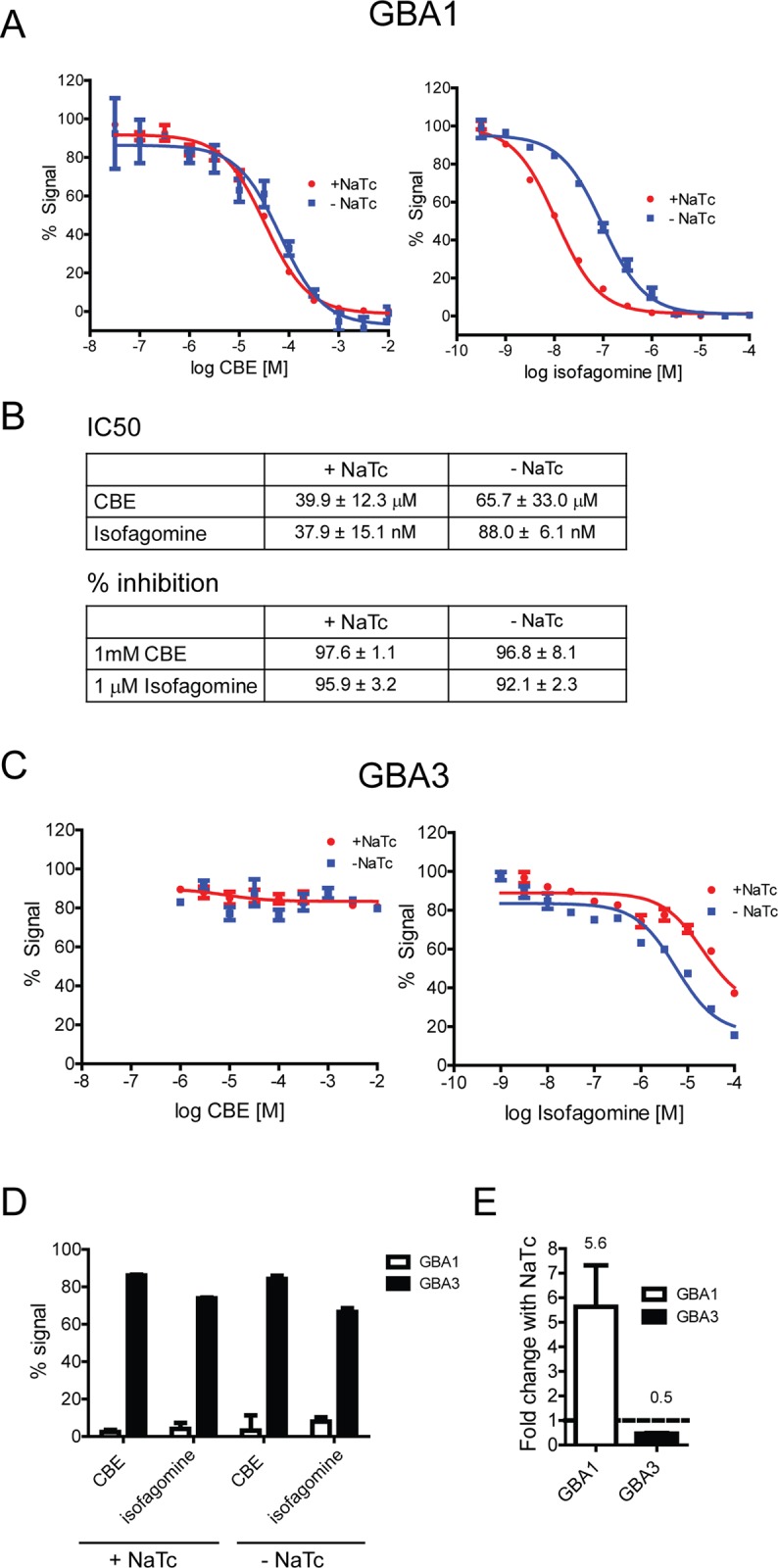
CBE and isofagomine effectively inhibit purified GBA1 at 1 mM and 1 μM, respectively. A) Dose response of GBA1 inhibition in a cell free system by CBE and isofagomine. Similar dose response curves are seen with and without sodium taurocholate (NaTc). A representative experiment is shown. B) IC_50_ values for isofagomine and CBE and % inhibition at 1 mM CBE and 1 μM isofagomine, respectively (mean ± SEM). Summary of data from three independent experiments is shown. C) CBE and isofagomine are not potent inhibitors of recombinant GBA3. A representative experiment is shown. D) CBE (1 mM) and isofagomine (1 μM) effectively inhibits GBA1 but not GBA3, irrespective of whether sodium taurocholate is present. The graph shows a summary of three independent experiments. E) Addition of sodium taurocholate (NaTc) increases activity of GBA1 ~ 5-fold but decreases activity of GBA3 by ~ 50%. Graph depicts a summary of three independent experiments.

We first determined the potency of two GBA1 inhibitors, CBE and isofagomine, under our conditions in a cell free system with purified human GBA1 and the artificial substrate 4MUG. We specifically tested conditions with and without sodium taurocholate, often used in similar cell free enzymatic assays to increase the assay window as a result of increased GBA1 activity. Sodium taurocholate did not have a dramatic effect on potency or efficacy of CBE and isofagomine (Fig. 1a, b). Importantly, 1 mM CBE or 1 μM isofagomine led to more than 90% inhibition of GBA1 activity (Fig. 1b).

In order to evaluate selectivity of CBE and isofagomine, we tested whether these compounds can affect enzymatic activity of other members of the GBA family, GBA2 and GBA3. CBE did not inhibit recombinant GBA3 (Fig. 1c) or GBA2 (S1 Fig.). While isofagomine inhibited recombinant GBA3, it was substantially less potent against GBA3 (~≥ 100-fold) than GBA1 (Fig. 1c). In fact, 1 μM isofagomine inhibited GBA1 by > 90% resulting in very low remaining assay signal, but the same isofagomine concentration did not have any substantial effect on GBA3 activity (Fig. 1d). Minimal effect of isofagomine on recombinant GBA2 was observed (S1 Fig.). Sodium taurocholate increased activity of GBA1 and decreased activity of GBA3 (Fig. 1e). However, sodium taurocholate did not alter the percent inhibition/signal with 1mM CBE and 1 μM isofagomine (Fig. 1d). In summary, 1 mM CBE and 1 μM isofagomine effectively inhibit GBA1 activity both in the presence and absence of sodium taurocholate and appear relatively specific to GBA1.

Recently, new small molecules were reported to increase GBA1 activity [19,20] in spleen homogenate containing mutant N370S glucocerebrosidase and the artificial substrate 4MUG. We first confirmed that two of these compounds, compounds 40 and 43 (S2a Fig.), also increased enzymatic activity of purified wild-type human GBA1 (S2b Fig.). This increase in activity in the 4MUG-based assay was not due to a fluorescent artifact as compounds 40 and 43 alone with 4MUG did not produce any dose dependent effect (data not shown). A smaller effect was seen when sodium taurocholate was present in the reactions (S2b Fig.), likely due to the fact that sodium taurocholate increased activity of GBA1 by itself (Fig. 1e), thus potentially lessening an additional increase. Indeed, we observed that increasing the amount of sodium taurocholate attenuated the effect of compound 43 (S2c Fig.). The maximal activation of GBA1 by compound 43 appeared greater than that by compound 40 in the absence of sodium taurocholate (S2b Fig.). Compounds 40 or 43 did not have any effect on the activity of recombinant GBA2 or GBA3 (S2d Fig.).

**Fig 2:**
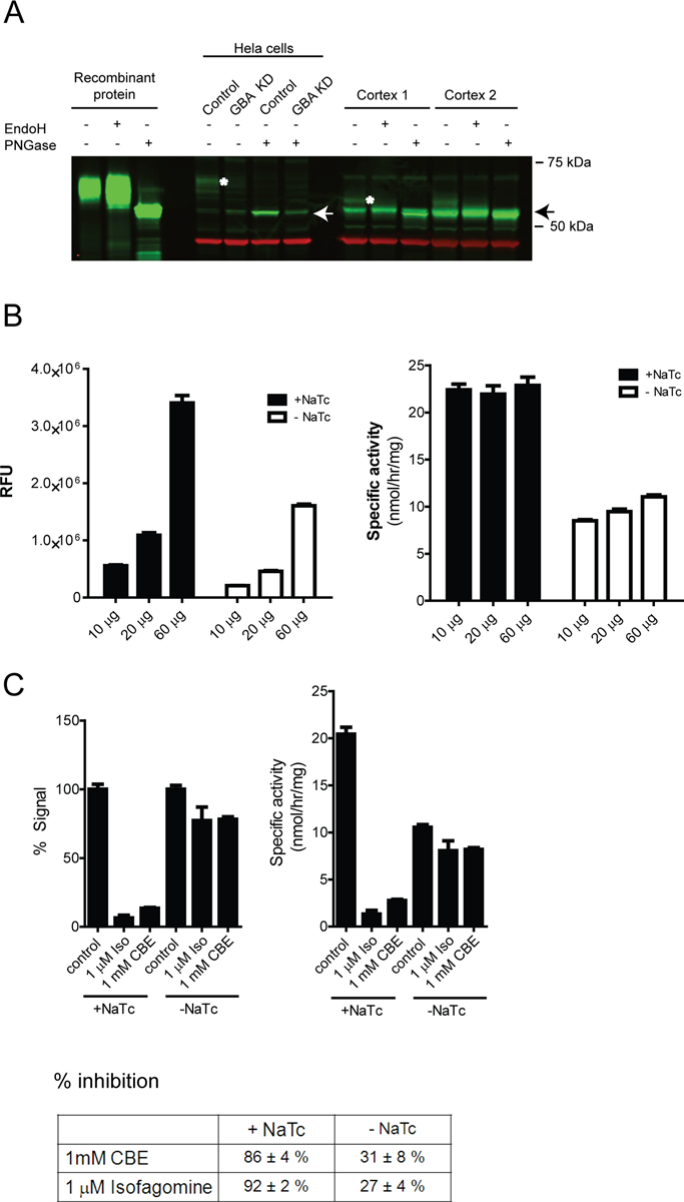
Turnover of 4MUG substrate reads out GBA1 activity in human brain lysates in the presence of sodium taurocholate. A) Glucocerebrosidase in human brain migrates at lower molecular weight compared to purified protein or GBA from Hela cells, likely due to lower glycosylation. This difference in migration is removed upon deglycosylation with PNGase. Asterisks denote migration of glycosylated glucocerebrosidase (in Hela cells and brain), arrows point to deglycosylated glucocerebrosidase protein. GBA KD – glucocerebrosidase knock-down with shRNA. B) Increasing amount of protein lysates in the activity assay leads to increased signal (RFU — relative fluorescence unit). When converted to specific activity, similar values are obtained when using 10–60 μg of human cortex lysates, as expected. A representative experiment is shown. C) Turnover of 4MUG substrate in lysates from human cortex is mostly due to GBA1 activity when using sodium taurocholate (NaTc). 1 mM CBE and 1 μM isofagomine (Iso) decrease the signal by ~ 90% in the presence of sodium taurocholate whereas substantially smaller reduction (~ 30%) is seen without sodium taurocholate in the assay buffer. 10 μg of protein lysate was used. A representative experiment is shown in the graphs, a summary of three experiments is depicted in the table (mean ± SEM). In the graph, control for each condition (with and without sodium taurocholate, NaTc) was set as 100%.

We observed that human GBA1 protein extracted from human cortex migrates at lower molecular weight on SDS-PAGE when compared to recombinant purified protein (Fig. 2a). This is likely due to lower glycosylation as this difference disappeared upon deglycosylation with PNGase (Fig. 2a). Since our interest is primarily in CNS disorders, we wanted to test if the less-glycosylated brain derived GBA1 could be also activated by compounds 40 and 43 using our 4MUG assay. We first established that increasing amounts of protein lysates lead to greater relative fluorescence units (RFU) and similar specific activity (nmol/hr/mg of protein, Fig. 2b), confirming linearity of our assay conditions. Similar results were observed with and without sodium taurocholate, with overall lower activity seen without sodium taurocholate (Fig. 2b), as expected from experiments with the cell free system (Fig. 1e).

Similar to previously published experiments [9,10,21,22], our assay in human brain lysates is based on turnover of an artificial substrate 4MUG. However, 4MUG can also be converted to fluorescence by other enzymes, including GBA2 and GBA3 (S1 and S2 Figs.). In order to test the specificity of 4MUG turnover (and our read-out) to GBA1 in human brain lysates, we used GBA1 specific inhibitors CBE and isofagomine at 1 mM and 1 μM, respectively. When using sodium taurocholate, turnover of 4MUG was inhibited by CBE and isofagomine by ~ 90%. Without sodium taurocholate, turnover of 4MUG was inhibited to a significantly smaller extent (~ 30%, Fig. 2c). This demonstrates that sodium taurocholate is necessary in order for the assay to predominantly read-out GBA1 activity in human brain lysates.

**Fig 3:**
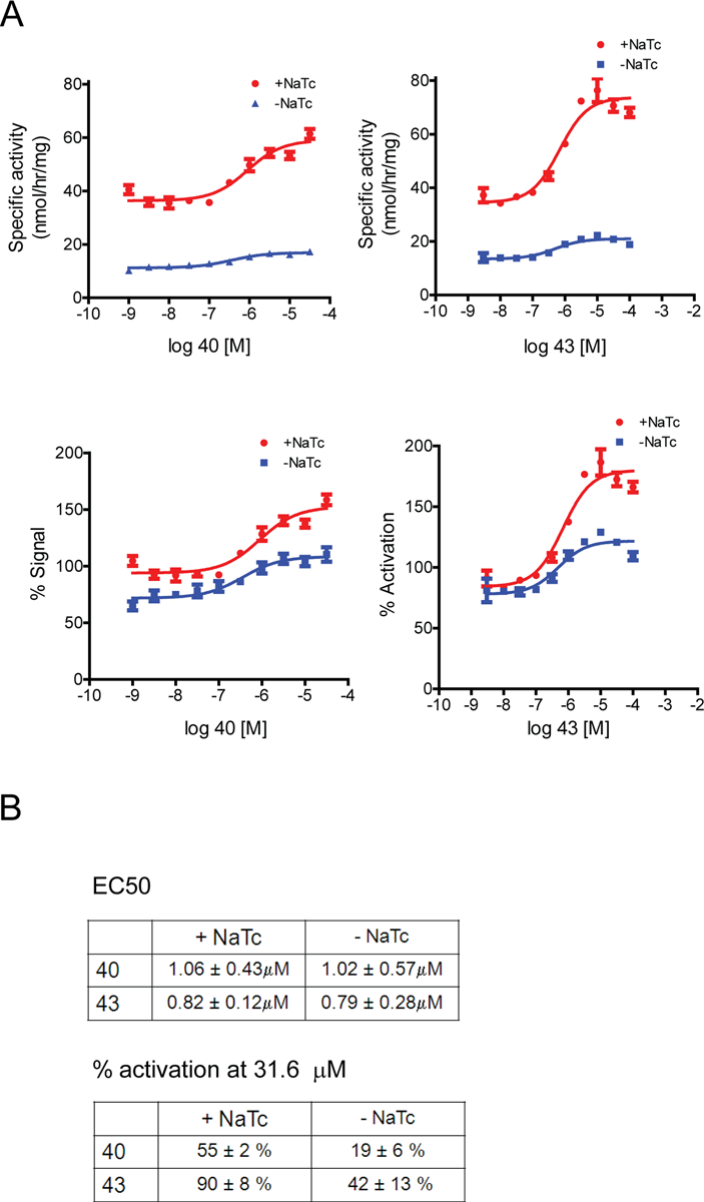
Tool compounds increase activity of human brain-derived glucocerebrosidase. A) Compounds 40 and 43 increase GBA1 activity in lysates from human cortex. In the graphs depicting % signal, vehicle treated samples were set as 100%. Representative experiments are shown. B) Table depicting average EC_50_ values and % activation from three independent experiments. Compound 43 activates GBA1 to a greater extent than compound 40 (90% vs 55%, respectively), based on activation at 31.6 μM, the highest concentration, at which both compounds were soluble.

Next, we tested whether tool compounds 40 and 43 can increase GBA1 activity in human brain lysates. While conditions with sodium taurocholate will read-out predominantly GBA1 activity, conditions without sodium taurocholate will read-out mostly the ability of other enzymes to turn over 4MUG (with ~ 30% contribution from GBA1). Compounds 40 and 43 led to a dose-dependent increase of GBA1 activity in the presence of sodium taurocholate (+NaTc) whereas a smaller increase was seen without sodium taurocholate (-NaTc) (Fig. 3a). This suggests that these compounds act selectively on GBA1 rather than non-specifically activating enzymes capable of 4MUG turnover. The EC_50_ for GBA1 (+NaTc) was 1.06 μM (compound 40) and 0.82 μM (compound 43). Compound 43 activated GBA1 to a greater extent compared to compound 40 (Fig. 3a, b), similar to our findings in a cell-free system (S2d Fig.). In summary, compounds 40 and 43 led to a robust increase in GBA1 activity in human brain lysates, suggesting that the lower glycosylation of brain GBA1 (compared to recombinant GBA1) did not affect compound binding.

**Fig 4:**
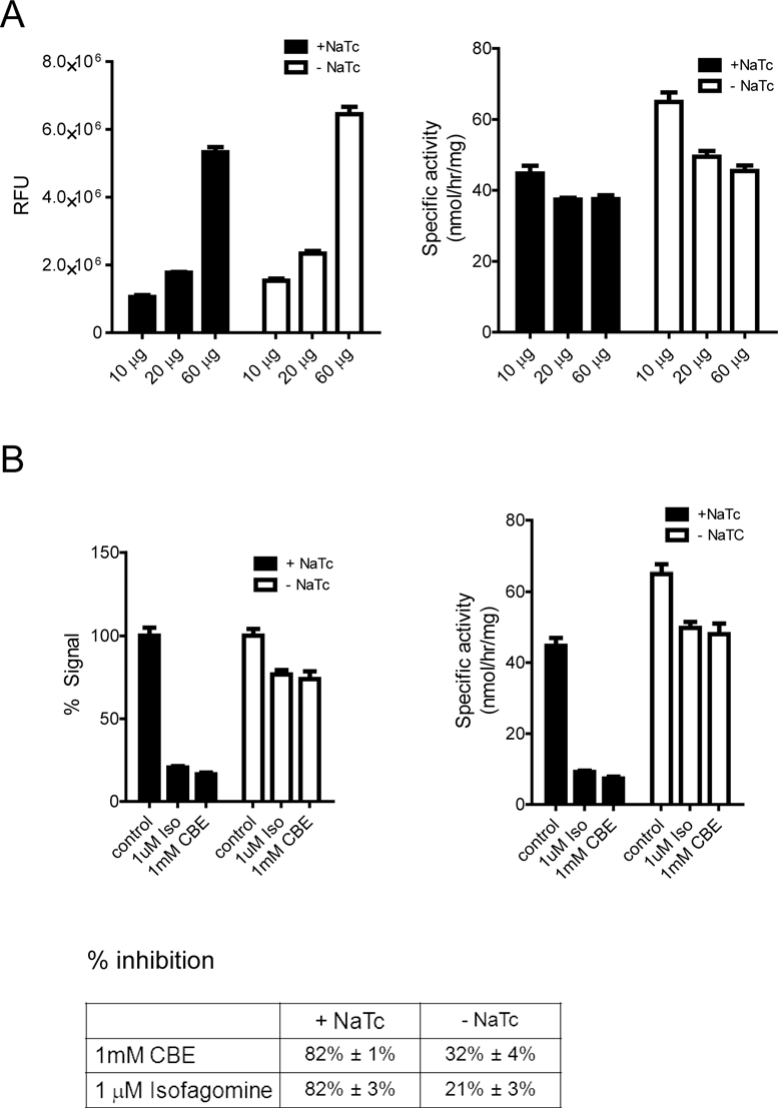
Turnover of 4MUG substrate detects GBA1 activity in mouse brain lysates in the presence of sodium taurocholate. A) Increasing amount of protein lysates in the activity assay leads to increased signal (RFU—relative fluorescence unit). When converted to specific activity, similar values are obtained when using 10–60 μg of mouse brain lysates, as expected. A representative experiment is shown. B) Turnover of 4MUG substrate in lysates from mouse brain is mostly due to GBA1 activity when using sodium taurocholate (NaTc). 1 mM CBE and 1 μM isofagomine (Iso) decrease the signal by ~ 80% in the presence of sodium taurocholate (+ NaTc) whereas substantially smaller reduction is seen without sodium taurocholate (- NaTc) in the assay buffer. 10 μg of protein lysate was used in the reaction. A representative experiment is shown in the graphs, a summary of three experiments is depicted in the table (mean ± SEM). In the graph, control for each condition (with and without NaTc) was set as 100%.

In order to test whether tool compounds can also activate mouse GBA, we first assessed specificity and linearity of the 4MUG assay using lysates from mouse brain. Increased amounts of lysate from mouse brain led to a higher signal and similar specific activity (Fig. 4a), similar to our results from human brain lysates. In contrast, addition of sodium taurocholate did not lead to an overall increase in activity as it did with the human brain lysates. GBA1 inhibitors CBE and isofagomine substantially decreased the assay signal only when sodium taurocholate was present (Fig. 4b), suggesting that the addition of sodium taurocholate is needed for this assay to primarily read out GBA1 activity, similar to our data in human brain lysates.

**Fig 5:**
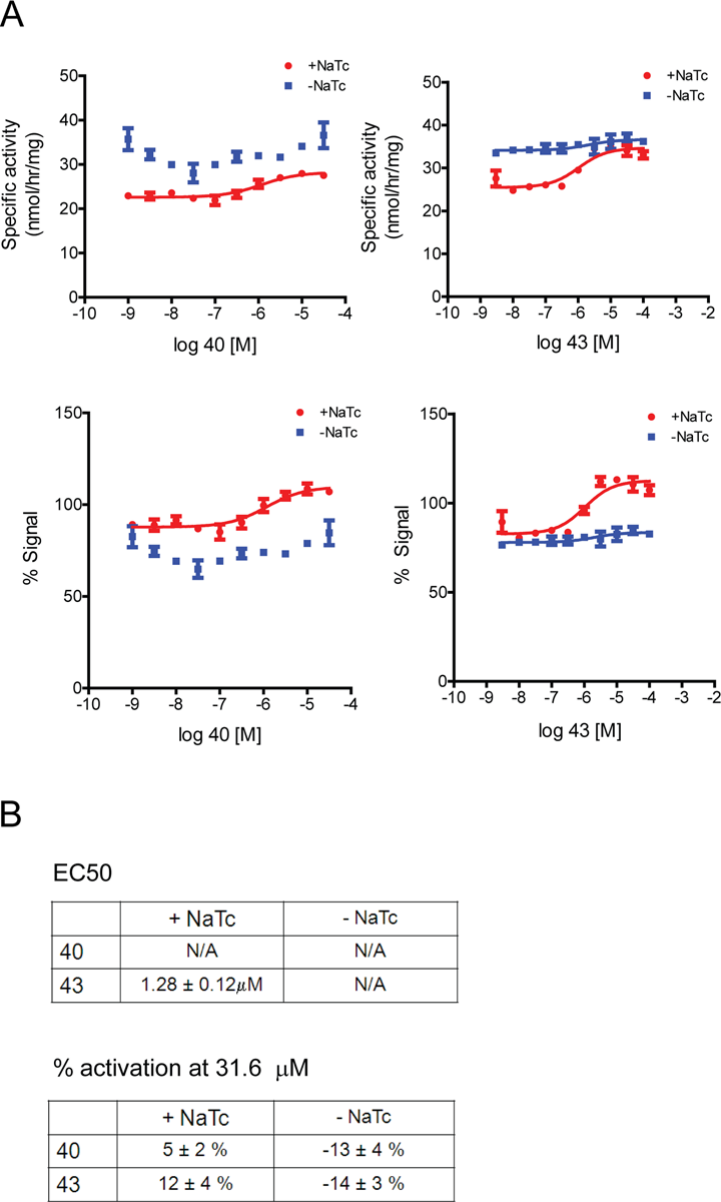
Tool compounds do not robustly increase activity of mouse brain derived GBA1. A) Compounds 40 and 43 do not robustly increase GBA1 activity in lysates from mouse brain. Subtle increases in activity were seen with sodium taurocholate (+NaTc) whereas subtle inhibition was observed without sodium taurocholate (-NaTc) upon compound addition. The inhibition was not dose dependent. Vehicle treated samples were set as 100% in the graph depicting % signal. Representative experiments are shown. B) Table depicting average EC_50_ values and % activation at the highest soluble concentration (31.6 μM) from three independent experiments.

**Fig 6:**
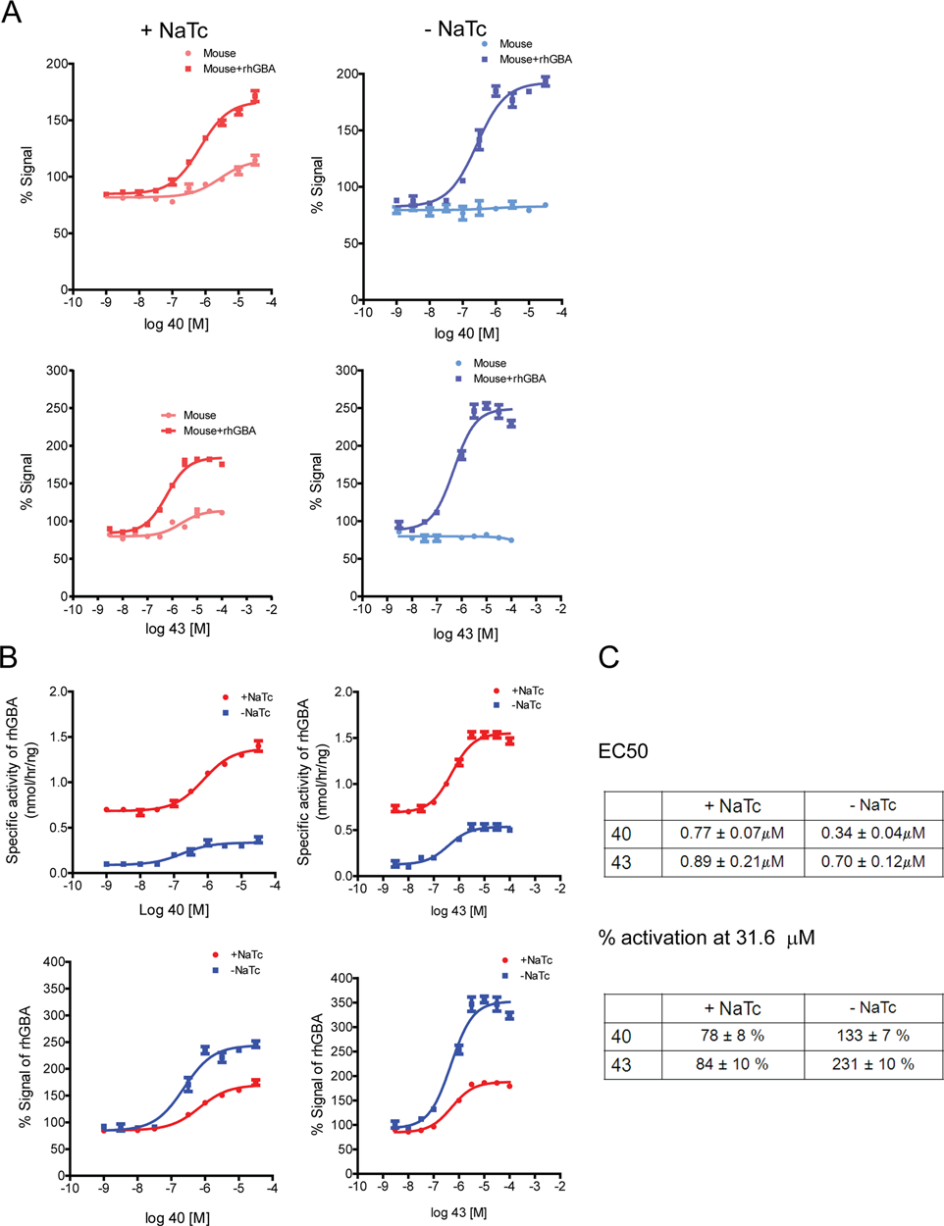
Tool compounds increase activity of human GBA1 when added into mouse brain lysates. A) Compounds 40 & 43 robustly increase activity of human purified GBA1 when added to mouse brain lysates (Mouse+rhGBA) while they do not substantially affect mouse GBA1 (Mouse), as measured by turnover of 4MUG substrate. Vehicle treated samples were set as 100%. A representative experiment is shown. B) Greater activation of human recombinant protein in mouse lysates is seen without sodium taurocholate. Activity of human recombinant protein in mouse lysates was obtained by subtracting values from a parallel experiment with mouse brain lysates but without added human recombinant protein. C) Table depicting EC_50_ values and % signal for purified human protein added to mouse brain lysates (three independent experiments). Mean ± SEM is shown.

Surprisingly, compounds 40 and 43 did not robustly modulate the activity of GBA1 in mouse brain lysates, although subtle effects were observed. When sodium taurocholate was added (GBA1-specific conditions), these compounds led to a very small increase in mouse GBA1 activity (~ 5%-12% on average, Fig. 5b). However, this small effect was highly variable and not seen in all experiments (data not shown). In contrast, in the absence of sodium taurocholate (non-specific 4MUG turnover), the tool compounds exhibited a small (~ 13%-14% on average) inhibitory effect (Fig. 5a, b), seen in all three experiments (data not shown). In summary, compounds 40 and 43 were found to robustly modulate GBA1 activity in human brain lysates but not in mouse brain lysates. A similar migration of human and mouse brain GBA1 was observed on SDS-PAGE (S6b Fig.). Post-mortem interval did not have any appreciable effect on GBA1 activity, both with and without compounds, and therefore is unlikely to account for the different responses to these tool compounds between mouse and human brain lysates (S3 Fig.).This unexpected difference between mouse and human brain lysates could be explained by either an inherent difference in GBA1 protein (mouse vs. human GBA1) or by the different tissue ‘environment’ (mouse vs. human brain). In order to test for the latter, i.e. whether mouse brain lysates could alter the efficacy of tool compounds, we added human recombinant GBA1 to the mouse brain lysates. Experiments were performed using an assay buffer with and without sodium taurocholate. Similar to previous experiments (Fig. 5), compounds 40 and 43 did not robustly modulate mouse GBA1 (Fig. 6a). However, a robust increase in 4MUG turnover was observed when human recombinant GBA1 protein was added to mouse brain lysates (Fig. 6a, b). This suggests that human GBA1 can be activated in mouse lysates and that the mouse lysates themselves did not prevent activity of these compounds.

**Fig 7:**
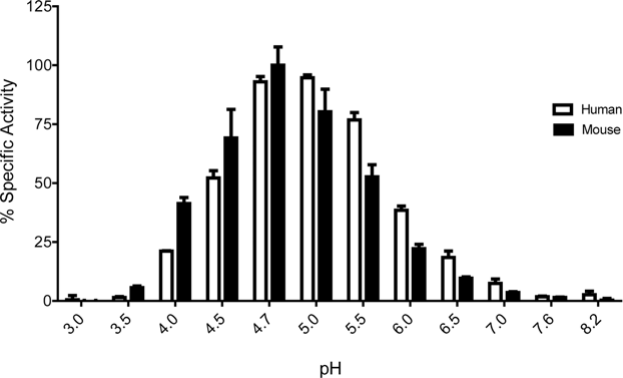
Mouse and human GBA1 exhibit maximal activity at similar pH. GBA1 in human and mouse brain lysates exhibit similar pH-dependent profile. Maximal activity of mouse GBA1 is seen at pH 4.7, maximal activity of human GBA1 is observed at pH 5.0 (value at pH 4.7 is only ~ 2% lower). Activity of mouse GBA1 at pH 4.7 was set as 100%. Sodium taurocholate was used.

These data raised the possibility that mouse GBA1 protein may differ from human GBA1 in many biochemical aspects. We decided to investigate one biochemical property, pH dependence of GBA1 enzymatic activity. This was previously reported to differ between wild-type and mutant glucocerebrosidase protein [20,23], which we also confirmed in our experiments (S4 Fig.). In order to test whether human and mouse GBA1 protein could exhibit similar pH-dependent differences, we compared GBA1 activity in human and mouse brain lysates (in the presence of sodium taurocholate). Human and mouse GBA1 exhibited similar specific activity and similar pH profile (Fig. 7) with maximum activity at pH 4.7–5.0, suggesting little difference between mouse and human GBA1 with respect to pH-dependence.

## Discussion

A recent publication showed that novel tool compounds 40 and 43 can increase turnover of 4MUG artificial substrate by the N370S mutant GBA1 protein in human spleen homogenates [19]. Our data show that these novel compounds increase 4MUG turnover by GBA1 derived from human cortex. The compound effects are seen despite differences in glycosylation between the recombinant and brain derived protein, implying that glycosylation is not important for compound binding. Surprisingly, we did not observe robust effects when assessing GBA1 enzymatic activity from mouse brain lysates. In order to determine whether this lack of effect was due to intrinsic differences between mouse vs. human brain environment or mouse vs. human GBA1 protein, human recombinant protein was added to mouse brain lysates. A strong GBA1 activation was then observed, suggesting that it is not the mouse brain environment but rather the different GBA1 protein sequence (S6 Fig.) accounting for this lack of effect.

We have not confirmed the lack of effect on mouse GBA1 in a cell free-system due to lack of commercially available recombinant mouse GBA1, which to our knowledge has not been previously purified. However, compounds 40 and 43 were able to increase turnover of 4MUG substrate from diverse sources of human GBA1 with different glycosylation patterns, including human recombinant protein (S2 Fig.), human brain (Fig. 3) and human spleen [19], suggesting that the source of GBA1 is not important for efficacy of these molecules. Therefore, we are not aware of any evidence which would suggest that these compounds would act differently on purified mouse GBA1 as opposed to mouse brain GBA1. Most importantly, the aim of our experiments was to assess compound effects in brain lysates with implications for suitable efficacy models. Thus, our data raise the possibility that mouse models with human GBA1 sequence may be needed in order to assess *in vivo* efficacy of small molecules aimed at modulating brain GBA1.

Our data also show that these compounds increase 4MUG turnover by recombinant GBA1 protein in a cell-free system without the need for the native co-factors, as originally suggested [20]. This suggests that these tool compounds could be potentially identified even using conventional HTS screening approaches with recombinant enzyme. We hypothesize that such approaches were previously not successful because these assays had been optimized for high GBA1 activity by adding sodium taurocholate [13]. Our data, showing that high sodium taurocholate concentrations in a cell free assay result in a lower GBA1 activation (S2c Fig.), are consistent with this hypothesis.

In our experiments with brain lysates, we report that the presence of sodium taurocholate is necessary in order for this assay to read-out specific GBA1 activity. Our data suggests that under these assay conditions, the read-out is at least ~ 90% and ~ 80% dependent on GBA1 in human and mouse brain lysates, respectively. Omitting sodium taurocholate from this reaction will read out activities of various enzymes capable of turning over the 4MUG substrate, with only a small contribution from GBA1. This data is important for future studies testing reproducibility of previously reported GBA1 deficits in human samples, where such analysis was not performed, making it difficult to assess how much of the signal is GBA1 dependent. Although several studies agree on differences in GBA1 activity in PD and DLB patients’ brain lysates [9–11], there are contradictory observations from human CSF [21,24–26], raising the possibility that assay conditions (such as sodium taurocholate) may explain these differences.

Since presence of sodium taurocholate in a cell-free assay makes it harder to see a compound effect, how is it then possible that we observed a robust increase of 4MUG turnover by GBA1 in human brain lysates containing sodium taurocholate? The most likely explanation is that a relatively small concentration of sodium taurocholate was used in our experiments with brain lysates. At this same final concentration of sodium taurocholate (3 mM), a robust (albeit smaller) increase in 4MUG turnover was seen even in a cell free system (S2 Fig.).

In a cell free system, compounds 40 and 43 increase 4MUG turnover to a greater extent without sodium taurocholate (S2 Fig.). This is consistent with the idea that further activating GBA1, which was already activated by sodium taurocholate (Fig. 1e), may be more difficult. This is also supported by our data in S2 Fig. showing that increasing concentration of sodium taurocholate in our recombinant assay leads to smaller increase of activity upon addition of tool compound 43. In contrast, these tool compounds exert a greater increase in 4MUG activity in human brain lysates when sodium taurocholate is used. We hypothesize that in tissues sodium taurocholate will lead this assay to be GBA1-specific. This is consistent with our results in Fig. 1, where we demonstrate that adding sodium taurocholate substantially decreases activity of GBA3. Thus, it is possible that without sodium taurocholate, other enzymes will contribute to 4MUG turnover to a greater extent, limiting the GBA1 contribution to the total assay signal. In summary, a small amount of sodium taurocholate in tissue lysates will allow the 4MUG readout to be primarily GBA1-dependent without hindering the ability of small molecules to activate it.

Our study relies on turnover of the 4MUG artificial substrate to assess GBA1 enzymatic activity, similar to many previous studies [9,10,21,22]. The advantages of this assay include its ease of use and high specificity to GBA1 in the presence of sodium taurocholate, even when using lysates containing many different lysosomal enzymes. While assays monitoring the turnover of natural substrate glucosylceramide have been described [27], these are limited to cell free systems and have not been commonly used for assessment of GBA1 in lysates. Nevertheless, it is important to emphasize that 4MUG is not a natural substrate, making it difficult to describe the tested molecules as ‘activators’. Indeed, we have not been able to detect increased turnover of the natural substrate, glucosylceramide, in a GBA1 cell free system with these compounds (S5 Fig.). It is worth noting, however, that this natural substrate assay represents a model system and does not recapitulate presence of the substrate in the lysosomal membrane. Nevertheless, our results are compatible with the initial publication [19] in which the tool compounds also did not have any effect on turnover of fluorescent ceramide in a spleen homogenate.

In summary, our data show that compounds 40 and 43 act robustly on glucocerebrosidase in human brain lysates but not on glucocerebrosidase in mouse brain lysates. This suggests that *in vivo* efficacy models with human glucocerebrosidase may be needed, such as the one recently reported by Sanders et al. [28].

## Methods

### GBA1 enzymatic cell-free assay

Recombinant human GBA1 was either purchased (R&D Systems, 7410-GH-10) or generated according to the protocol below (see purification of recombinant glucocerebrosidase). No differences were observed between commercial and internally generated GBA1 when testing compounds (data not shown). GBA3 (R&D Systems, 5969-GH-10) and GBA2 (Abcam, ab163631) were purchased. GBA1 and GBA3 enzymes were diluted to 0.3 ng/μl in homogenization buffer (250 mM Sucrose, 10 mM Tris pH = 7.5, 1 mM EDTA, 0.25% Triton X-100 solution) containing protease inhibitors (Sigma, P8340), phosphatase inhibitors I and III (Sigma, P2850, P0044), at 1:100 dilution, and PMSF, at 1:1,000 dilution. 7.5ng recombinant GBA1 (final concentration 0.87 nM) was added to black 96-well plates (Costar, 7200567) in 25 μl volume, followed by addition of compounds (when appropriate, 1.5 μl), and incubated for 30 minutes on ice.

After incubation, 100 μl of assay buffer was added to the plate. The assay buffer was composed of 0.1 M citric acid and 0.2 M sodium phosphate dibasic adjusted to pH = 5.4 unless stated otherwise (McIlvaine Buffer). Assay buffer was used with and without sodium taurocholate (Sigma, T4009) at a concentration 4.65 mM (final concentration 3.1 mM). For IC50 and EC50 experiments using compounds an assay buffer of pH 5.4 was used.

The substrate, 4-methylumbelliferyl β-D-glucopyranoside (4MUG, Sigma, M3633) was then added to the plate to a final concentration of 1.7 mM (25 μl). 4MUG was freshly solubilized in water at 37°C prior to each experiment. The recombinant protein, compound, assay buffer, and substrate were incubated at 37°C for 30 minutes. 75 μl stop solution (1 M Glycine adjusted with 1M NaOH to pH = 10.5), was added to the plate. The fluorescent signal was detected using the EnVision Multilabel Plate Reader (Perkin Elmer, excitation = 355, emission = 450).

The standard used for this assay was a fluorescent product 4-Methylumbelliferone (4MU) (Sigma, M1381), solubilized in DMSO, at 26.56 μM, 8.85 μM, 2.95 μM, 0.98 μM, 0.33 μM, and 0.11 μM final concentration. The specific activity was calculated off the 4MU standard curve by converting the relative fluorescence units (RFUs) to the concentration of the fluorescent product (GraphPad Prism 5.1). This interpolated value was then used to calculate the specific activity of the recombinant protein which was expressed as nmol/hr/ng protein for recombinant protein and nmol/hr/mg for tissue lysates (GBA1 tissue assay). The standard curve was always diluted in the same assay buffer as the samples, including the same pH. For experiments testing activity of GBA1 at different pH, a standard curve for each pH condition was used to calculate specific activity. When testing different pH conditions, buffer solutions were adjusted to obtain the desired pH using the McIlvaine’s Buffer recipe, and the pH of the buffer was verified. All assay buffers used in the pH experiments contained sodium taurocholate to a final concentration of 3.1 mM.

In experiments directly comparing enzymatic activities of GBA1, GBA2 and GBA3 (S1 Fig., S2 Fig. c, d) the following conditions were used. The assay buffer consisted of 44 mM Citric Acid, 111 mM Na_2_HPO_4_, pH 5.5, 0.1% Triton X-100. Internally purified GBA1, GBA2 (Abcam, ab163631) and GBA3 (R&D Systems, 5969-GH-010) were diluted to 0.24 ng/μl, 4.2 ng/μl and 0.02 ng/μl respectively. A 10-fold intermediate solution of compound serial dilution was prepared in an assay buffer; half log dilutions were used. In a black 384-well plate (ThermoFisher, 267461) we added 5 μl of enzyme solution and 1 μl of compounds when appropriate, and incubated for 30 minutes at room temperature. 2 mM solution of 4MUG was prepared in an assay buffer. To start the reaction, 4.5 μl/well of this solution was added to the enzyme plate and was incubated for 30 minutes at 37°C. To stop the reaction, a 1M glycine solution, adjusted to pH = 10 using 1M NaOH was used. The fluorescent signal was detected using the Envision Multilabel Plate Reader (Perkin Elmer) at excitation = 355nm and emission = 450nm. To test the effect of sodium taurocholate (Sigma, T4009), the assay was performed using the same procedure described in this paragraph. The assay buffer contained either 0mM (same as above), 3 mM, 7 mM or 20 mM Sodium Taurocholate.

### GBA1 tissue assay

Brain lysates were prepared by mechanical homogenization of mouse brain and human cortex in 10x weight/volume of the homogenization buffer used in the GBA1 enzymatic cell-free assay (250 mM Sucrose, 10 mM Tris pH = 7.5, 1 mM EDTA, 0.25% Triton X-100 solution, protease inhibitors, phosphatase inhibitors I and III, PMSF). Human cortex was acquired from brain banks (Tissue Solutions: http://www.tissue-solutions.com; Proteogenex: http://www.proteogenex.com). Human cortex samples were obtained from deceased aged Caucasian subjects without any diagnosis of neurodegenerative disorders. Mouse brain was obtained from 4–7 month old C57BL/6J mice. All mouse experiments were performed in accordance with the specifications of both the National Institutes of Health Guide for the Care and Use of Laboratory Animals, studies were approved by Pfizer’s Internal Animal Care and Use Committee (IACUC).

Prior to 30 min lysis on ice, human brain samples were also sonicated for 15 seconds as this step was needed to fully extract GBA1 (data not shown). Samples were then centrifuged at 20,000g for 15 minutes at 4°C. Supernatant was collected, and used for activity assays and Western blots. Protein concentration was determined using the Pierce BCA protein assay kit. Tissue lysates and pellets were stored at -80°C until use. For enzymatic activity assays, lysates were subjected only to one free-thaw cycle. Independent replicates were obtained using brain lysates from either different human subjects or different mice.

GBA1 activity in tissues was measured using the assay conditions described in the previous section. 10 μg of protein/well was used for the activity experiments, unless stated otherwise. Brain tissue lysates were diluted to 0.4 μg/ μl of protein in the same homogenization buffer, as described above. 25 μl of diluted lysate was added to the plate (Costar, 7200567). Lysates were then incubated on ice in the presence or absence of a compound for 30 minutes. 100 μl of assay buffer was then added to the plate. The same assay buffer was used as in the cell free/recombinant experiments and processed as described above.

For the activity experiments combining mouse brain tissue lysates and human recombinant GBA protein, tissue lysates were diluted to 0.8 μg/μl and 12.5 μl of sample was added to plate to a final concentration of 10 μg of protein/well. The recombinant WT GBA1 was diluted to 0.6 ng/μl, and 12.5 μl of sample was added to the plate for a final concentration of 7.5 ng/well. All other assay conditions were identical as described above. To calculate the specific activity in this experiment, the fluorescent signal from the dose response curve with mouse tissue lysates alone was subtracted from the fluorescent signal of the recombinant protein in the presence of the mouse tissue lysates, and was calculated as described above.

### Western Blot

Samples were digested using EndoH and PNGase F (New England Biolabs, P0702S & P0704S) according to New England Biolabs protocol. Briefly, protein samples were denatured for 10 minutes at 100°C, deglycosylated for 2 hours at 37°C, and then the reaction was stopped for 10 minutes at 75°C. Samples were stored at-80°C until use. GBA knockdown cells were generated using Sigma MISSION shRNA lentivirus particles (Sigma, TRCN0000029298).

Samples (~ 30–40 μg of protein lysates, ~ 10–50 ng for recombinant GBA protein) were separated using 10% Tris-glycine gel (Life Technologies, WT0102A), then transferred for 8 minutes onto nitrocellulose membranes using the iBlot Gel Transfer Device (Life Technologies). Membranes were incubated with primary antibodies, anti-glucocerebrosidase c-terminal antibody (Sigma, G4171, 1:1,000) and monoclonal anti-actin (Sigma, A2228, 1:80,000) overnight at 4°C. Membranes were then washed, and incubated with secondary antibodies, goat anti-rabbit (1:10,000) and goat anti-mouse (1:40,000) (LI-COR). Western blots were developed, and analyzed using the Odyssey infrared scanning system (LI-COR).

### Purification of recombinant glucocerebrosidase

Wild type human glucocerebrosidase was first cloned into a pFastBac-1 vector (Invitrogen) using standard molecular biology techniques and subsequently used to generate recombinant baculovirus using the Invitrogen Bac-to-Bac system. Viral amplification was perfomed in SF-9 cells and protein expression occurred in either SF-9 or SF-21 cells. Conditioned media was harvested 72 hours post-infection by centrifugation and filtered (0.22 μm). Protein was purified by overnight batch binding to Toyopearl Butyl-650C resin (Hydrophobic Interaction Chromatography) in conditioned media supplemented with 500 mM NaCl. Protein was eluted with 20 mM sodium acetate pH 5.0, 150 mM NaCl, 50% ethylene glycol. Ethylene glycol was removed by dialysis, and protein was further purified by size-exclusion chromatography (Superdex200) into 25 mM Tris pH 7.25, 120 mM NaCl, 0.5 mM TCEP, 5% Glycerol. N370S mutant glucocerebrosidase construct was generated by Quikchange site-directed-mutagenesis (Agilent Technologies). Virus generation, amplification, and protein expression for N370S were performed as described above for the wild-type protein. N370S was purified by batch binding to Toyopearl Butyl-650C resin followed by binding to a HiTrap Heparin column and eluted into 20 mM sodium acetate pH 5.0, 20% ethylene glycol with a linear gradient of sodium chloride.

Generation of de-glycosylated glucerebrosidase was based on previous report [29]. In summary, GCase (prepared as described above) was concentrated to 0.7 mg/mL and dialyzed overnight at 4°C against PBS at pH 7. PNGase F (New England Biolabs P0704S) was added at 20 units/mg glucorebrosidase and incubated ~3 days at room temperature with provided reaction buffer. Reaction components were removed by size-exclusion chromatography (SD200) to quench the reaction. Deglycosylation was confirmed by changed migration on western blot. The extent of the migration difference on SDS-PAGE suggested that partial deglycosylation likely occurred using this established protocol, potentially due to inaccessibility of some glycosylation sites without full denaturation of the protein.

### Assay measuring turnover of glucosylceramide

5 nM wild-type human GBA1 protein was incubated with varying amounts of glucosylceramide (Avanti Polor Lipids, #860547P) for various time points in reaction buffer containing Na_2_HPO_4_, 2.5 μg/ml Saposin C, 2.5 μg/ml Phosphatidylserine, 0.1% Triton X-100, adjusted to pH 5.0 with citric acid. At the end of each time point the reaction was terminated and the amount of glucose formed was determined using an equal amount the Amplex Red Glucose/Glucose Oxidase Assay Kit from Invitrogen (A22189) consisting of 100 μM Amplex Red reagent, 0.2 U/ml HRP, 2 U/ml glucose oxidase and 20 μM Isofagomine. For testing compounds, 0.75 nM glucocerebrosidase protein was incubated with 50 μM glucosylceramide (Avanti Polor Lipids, #860547P) for 30 minutes at room temperature in the same buffer, as described above. Glucose levels were determined using the Amplex Red Glucose/Glucose Oxidase Assay Kit from Invitrogen (A22189), as described above.

## Supporting Information

S1 Fig CBE and isofagomine do not act as potent inhibitors of GBA2 or GBA3. CBE inhibits GBA1 and does not alter activity of GBA2 or GBA3 within the concentrations tested. Isofagomine acts as a potent inhibitor of GBA1 while it is substantially less potent (> ~ 100-fold) against GBA3 and has almost no effect on GBA2 activity. 1 mM CBE or 1 μM isofagomine robustly inhibit GBA1 but not GBA2 or GBA3. A representative experiment is shown.
(TIF)

S2 Fig Tool compounds increase activity of human purified GBA1 but not GBA2 or GBA3 in a cell free system. A) Structures of compounds 40 & 43. B) Compounds 40 and 43 increase activity of purified GBA1 in a cell free system. Greater activation is seen with compound 43 (370% signal) compared to compound 40 (210% signal). EC_50_ for compound 40: 0.79 μM (+NaTc), 0.23 μM (-NaTc), EC_50_ for compound 43: 0.87 μM (+NaTc); 0.43 μM (-NaTc). A representative experiment is shown. C) Higher concentration of sodium taurocholate (NaTc) leads to lower activation of GBA1 in the presence of compound 43. A representative experiment is shown. D) Compounds 40 and 43 do not alter activity of GBA2 or GBA3. A representative experiment is shown.
(TIF)

S3 Fig Post-mortem interval does not substantially affect GBA1 activity with or without a tool compound. A) Incubating mouse lysates at room temperature (to mimic human post-mortem interval) does not lead to a substantial change in compound 43 efficacy. Data in the graph depict GBA1 activity in the presence of compound 43 (10 μM), relative to GBA1 activity in the presence vehicle/DMSO (set as 100% for each condition). Prior to performing GBA1 4-MUG activity assay, mouse brains were extracted and left at room temperature for 0, 2, 4 or 24 hours to resemble human post-mortem interval. Compound 43 (10 μM) consistently showed only a small effect in mouse brain lysates. Human cortex lysate (4hrs post-mortem interval) was included as a control. The graph represents a summary of three independent experiments using four mice per group (mean ± SEM). B) GBA1 activity in human brain lysates does not show an obvious correlation with a post-mortem interval. GBA1 activity without any compound is shown. C) In human brain lysates, post-mortem interval does not show an obvious correlation to either potency or efficacy of compounds 40 and 43. % Signal represents signal at compound concentration of 31.6 μM.
(TIF)

S4 Fig Wild-type and N370S mutant GBA1 protein exhibit different pH optima. Recombinant human wild-type protein has maximal activity at pH 5.0 whereas mutant N370S protein exhibits pH optimum at pH 6.0. N370S protein is substantially less active compared to wild-type protein. Activity values are expressed per the same amount of wild-type and N370S mutant GBA1 protein. Activity of wild-type protein at pH 4.7 was set as 100%.
(TIF)

S5 Fig Measuring activity of GBA1 using turnover of glucosylceramide. A) Assay measuring turnover of glucosylceramide shows Michaelis-Menten kinetics, typical for enzymatic reactions. B) Glucose standard curve showing linearity of the assay. Based on RFUs of approximately 6x10^5^ seen in our experiments, the formation of glucose showed ~ 13% turnover of substrate, compatible with detecting enhancers of GBA1 activity under these conditions. C) Compounds 40 and 43 do not increase human GBA1 activity when measured by cleavage of glucosylceramide substrate.
(TIF)

S6 Fig Comparison of mouse and human GBA1 protein. A) Sequence alignment of human and mouse GBA1 proteins. Positions of conservation are indicated by red bars and numbering corresponds to human GBA1. Overall, sequence identity is ~87%. B) Glucocerebrosidase from human and mouse brain migrates at a similar molecular weight on SDS-PAGE/western blot, suggesting a similar extent of glycosylation. Green–GBA1, red–actin.
(TIF)

S7 Fig Activity of human recombinant GBA 1, which underwent deglycosylation or sonication, can be also modulated by compound 43. 10 μM compound 43 was used, recombinant GBA1 was sonicated prior to performing the 4MUG enzyme activity assay.
(TIF)
