# Supplementary figures and images for: Tool Compounds Robustly Increase Turnover of an Artificial Substrate by Glucocerebrosidase in Human Brain Lysates
Source: PLoS One. 2015 Mar 12;10(3):e0119141. doi: 10.1371/journal.pone.0119141 (PMC4357465; doi:10.1371/journal.pone.0119141)

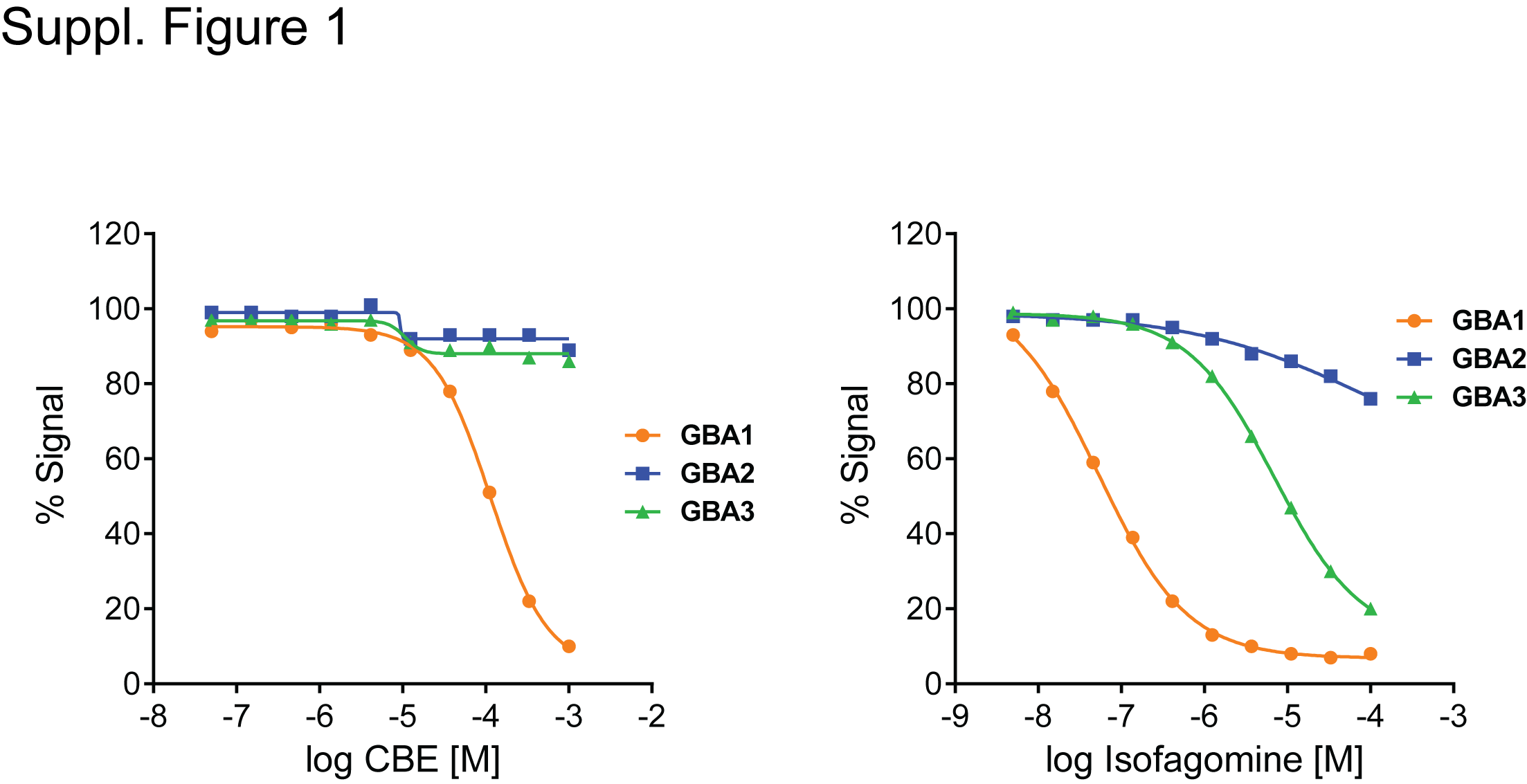

Supplement: S1 Fig — CBE inhibits GBA1 and does not alter activity of GBA2 or GBA3 within the concentrations tested. Isofagomine acts as a potent inhibitor of GBA1 while it is substantially less potent (> ~ 100-fold) against GBA3 and has almost no effect on GBA2 activity. 1 mM CBE or 1 μM isofagomine robustly inhibit GBA1 but not GBA2 or GBA3. A representative experiment is shown. (TIF) [file pone.0119141.s001.tif]

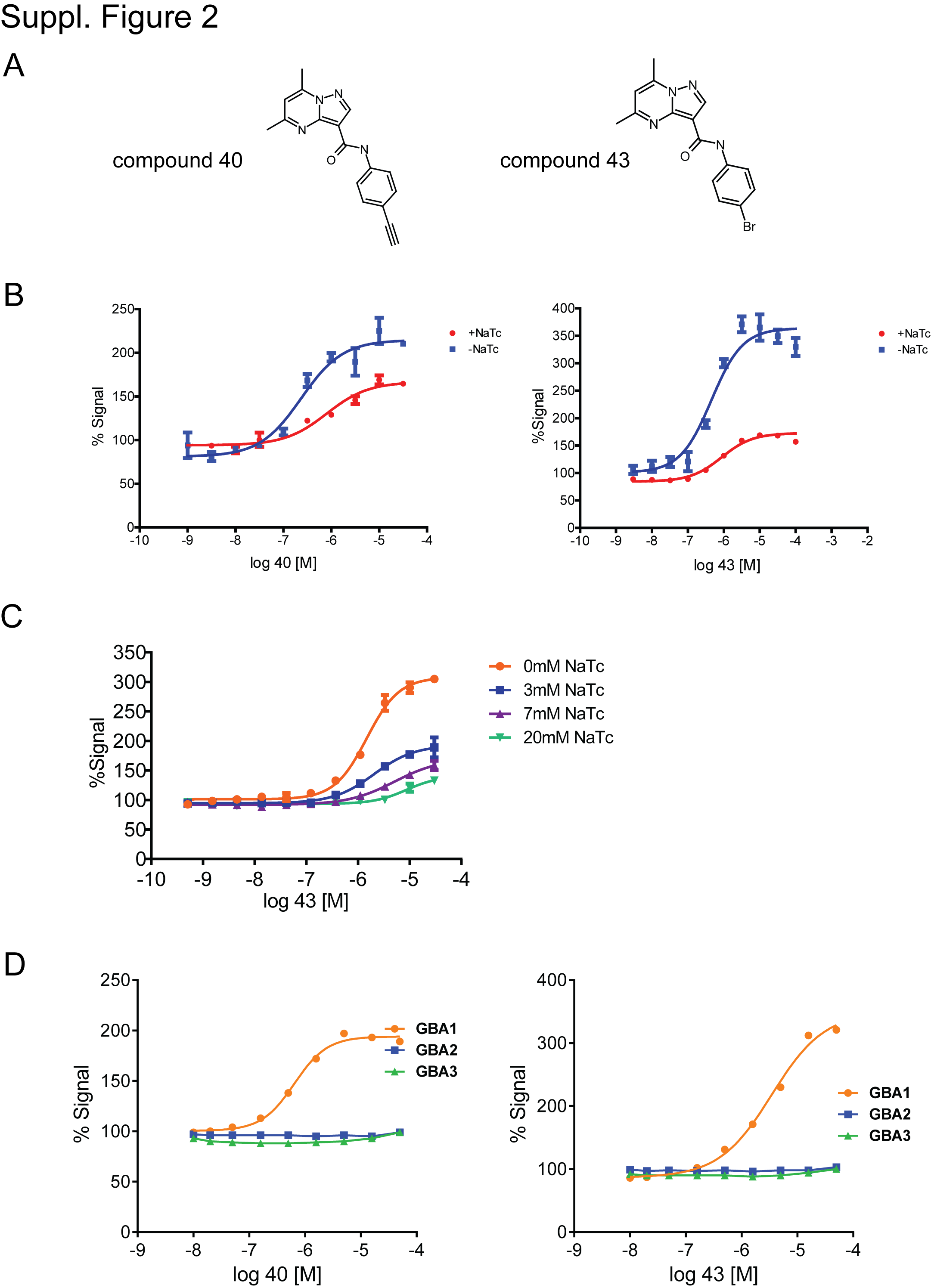

Supplement: S2 Fig — A) Structures of compounds 40 & 43. B) Compounds 40 and 43 increase activity of purified GBA1 in a cell free system. Greater activation is seen with compound 43 (370% signal) compared to compound 40 (210% signal). EC50 for compound 40: 0.79 μM (+NaTc), 0.23 μM (-NaTc), EC50 for compound 43: 0.87 μM (+NaTc); 0.43 μM (-NaTc). A representative experiment is shown. C) Higher concentration of sodium taurocholate (NaTc) leads to lower activation of GBA1 in the presence of compound 43. A representative experiment is shown. D) Compounds 40 and 43 do not alter activity of GBA2 or GBA3. A representative experiment is shown. (TIF) [file pone.0119141.s002.tif]

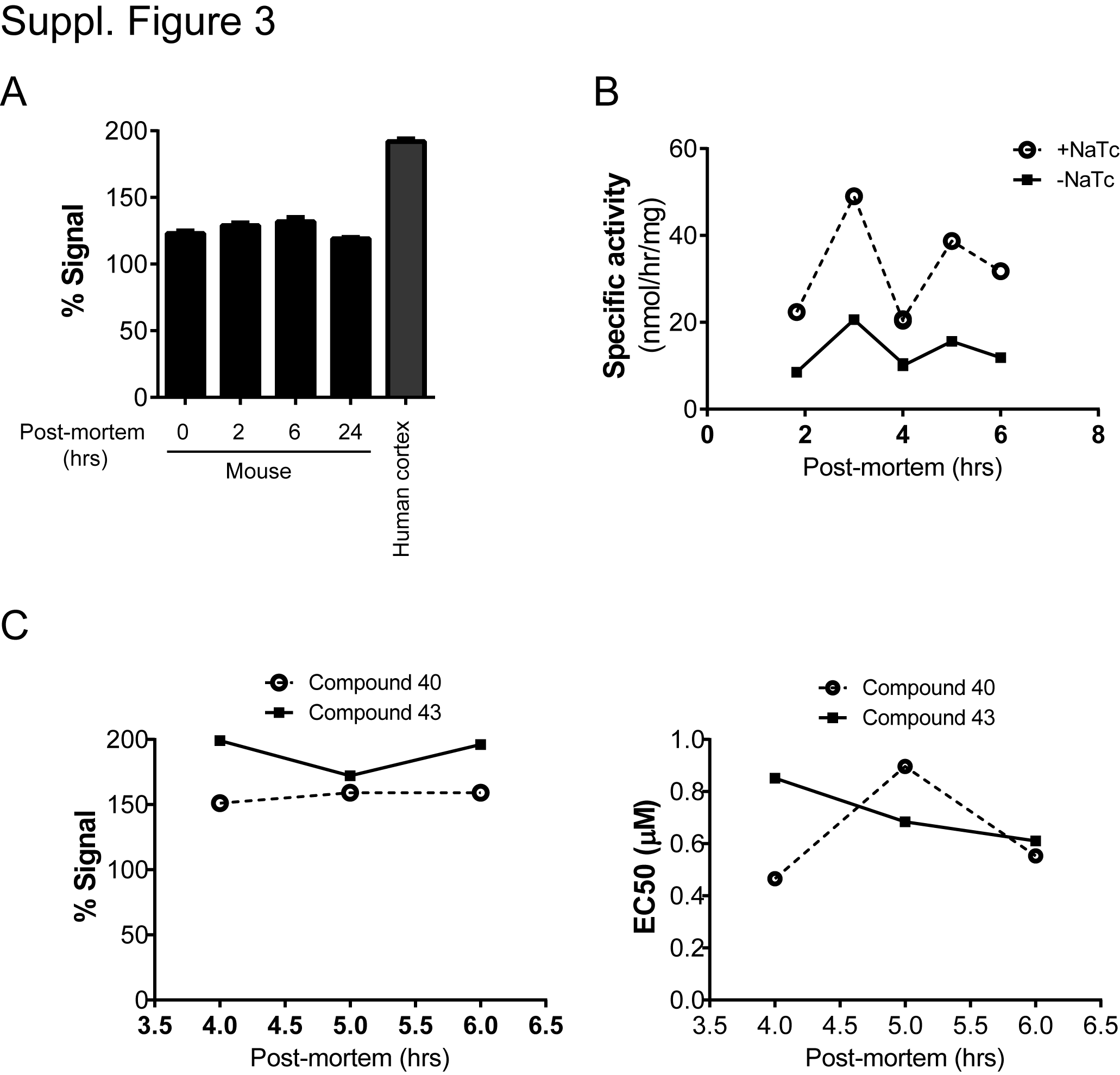

Supplement: S3 Fig — A) Incubating mouse lysates at room temperature (to mimic human post-mortem interval) does not lead to a substantial change in compound 43 efficacy. Data in the graph depict GBA1 activity in the presence of compound 43 (10 μM), relative to GBA1 activity in the presence vehicle/DMSO (set as 100% for each condition). Prior to performing GBA1 4-MUG activity assay, mouse brains were extracted and left at room temperature for 0, 2, 4 or 24 hours to resemble human post-mortem interval. Compound 43 (10 μM) consistently showed only a small effect in mouse brain lysates. Human cortex lysate (4hrs post-mortem interval) was included as a control. The graph represents a summary of three independent experiments using four mice per group (mean ± SEM). B) GBA1 activity in human brain lysates does not show an obvious correlation with a post-mortem interval. GBA1 activity without any compound is shown. C) In human brain lysates, post-mortem interval does not show an obvious correlation to either potency or efficacy of compounds 40 and 43. % Signal represents signal at compound concentration of 31.6 μM. (TIF) [file pone.0119141.s003.tif]

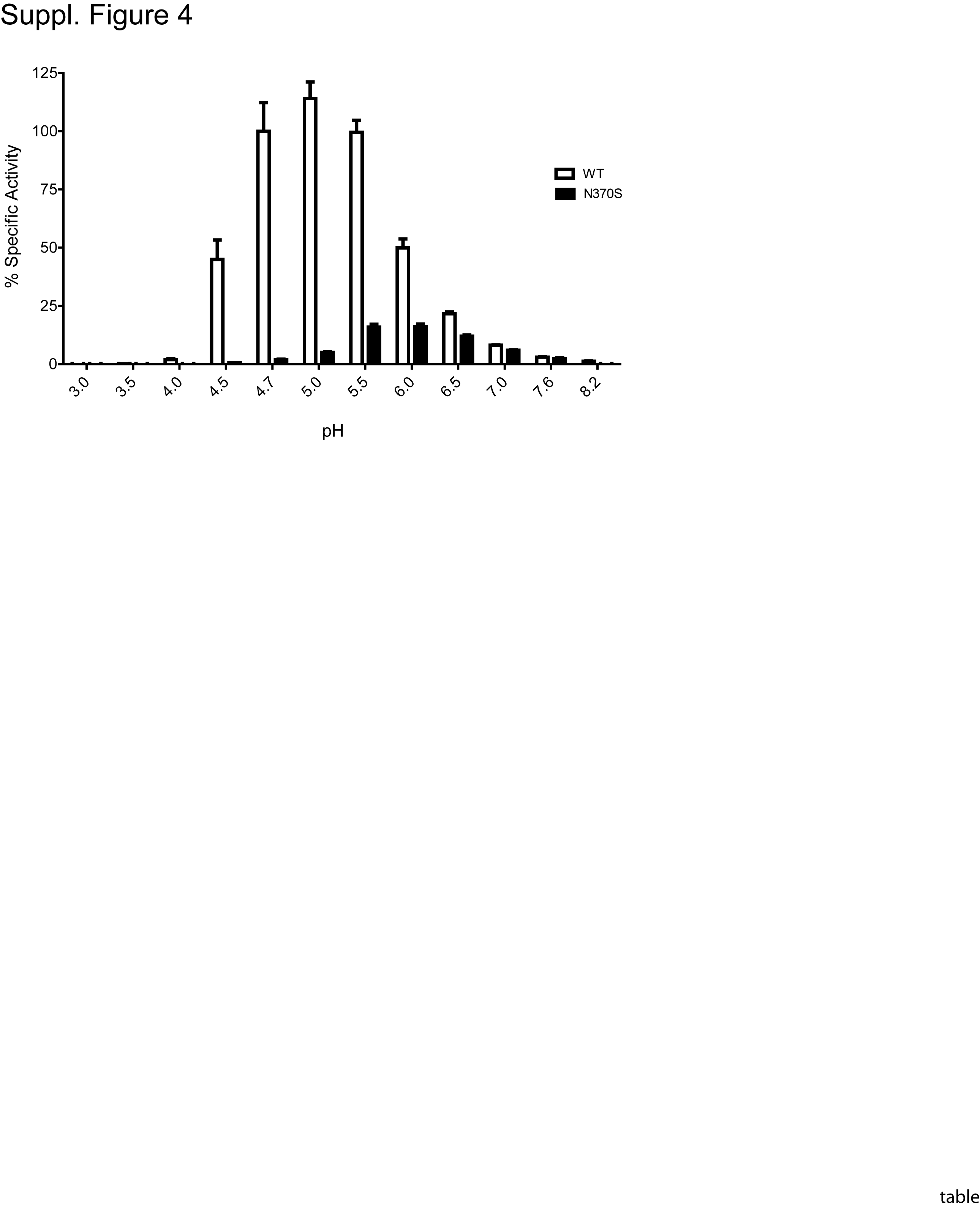

Supplement: S4 Fig — Recombinant human wild-type protein has maximal activity at pH 5.0 whereas mutant N370S protein exhibits pH optimum at pH 6.0. N370S protein is substantially less active compared to wild-type protein. Activity values are expressed per the same amount of wild-type and N370S mutant GBA1 protein. Activity of wild-type protein at pH 4.7 was set as 100%. (TIF) [file pone.0119141.s004.tif]

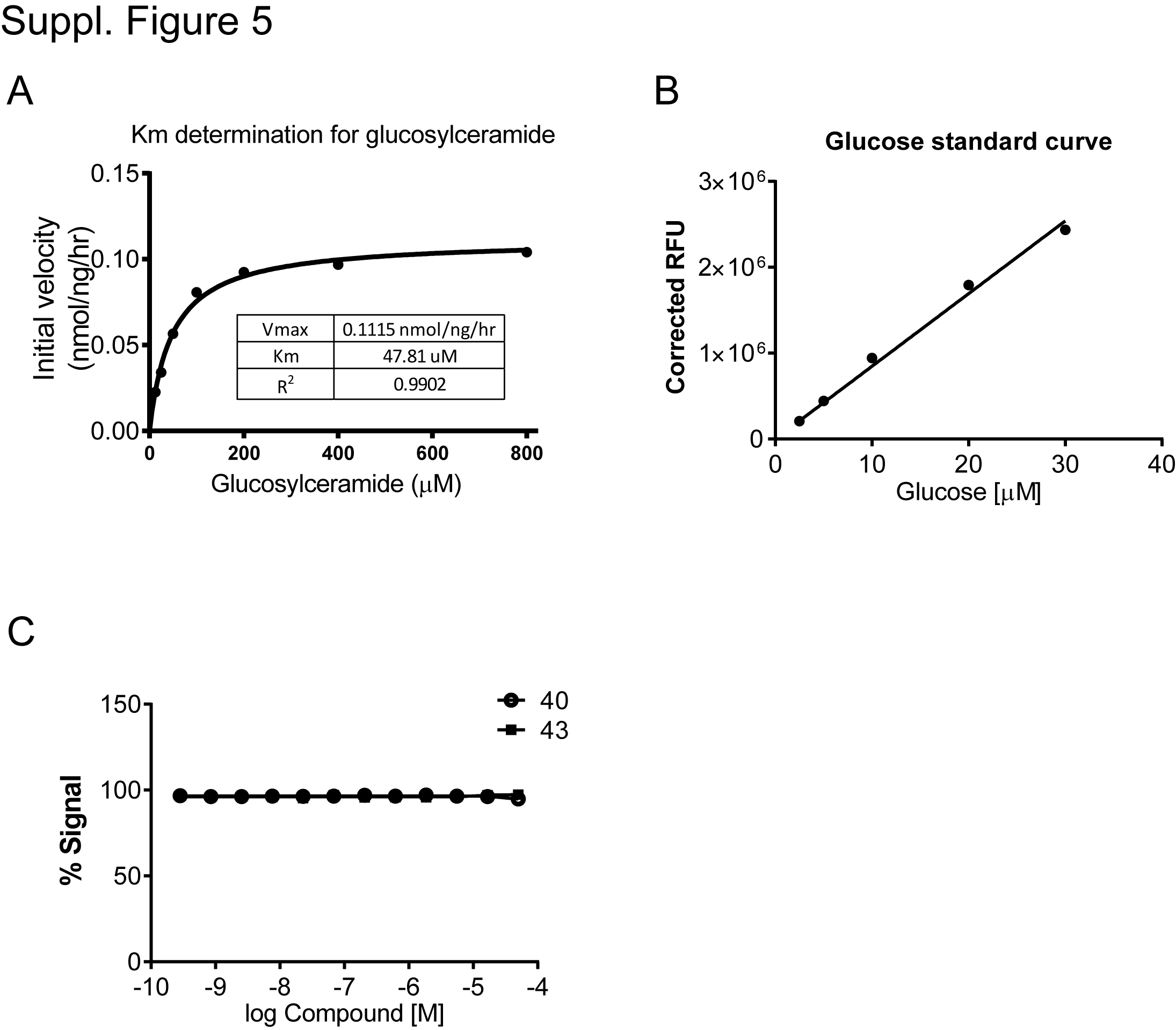

Supplement: S5 Fig — A) Assay measuring turnover of glucosylceramide shows Michaelis-Menten kinetics, typical for enzymatic reactions. B) Glucose standard curve showing linearity of the assay. Based on RFUs of approximately 6x10^5^ seen in our experiments, the formation of glucose showed ~ 13% turnover of substrate, compatible with detecting enhancers of GBA1 activity under these conditions. C) Compounds 40 and 43 do not increase human GBA1 activity when measured by cleavage of glucosylceramide substrate. (TIF) [file pone.0119141.s005.tif]

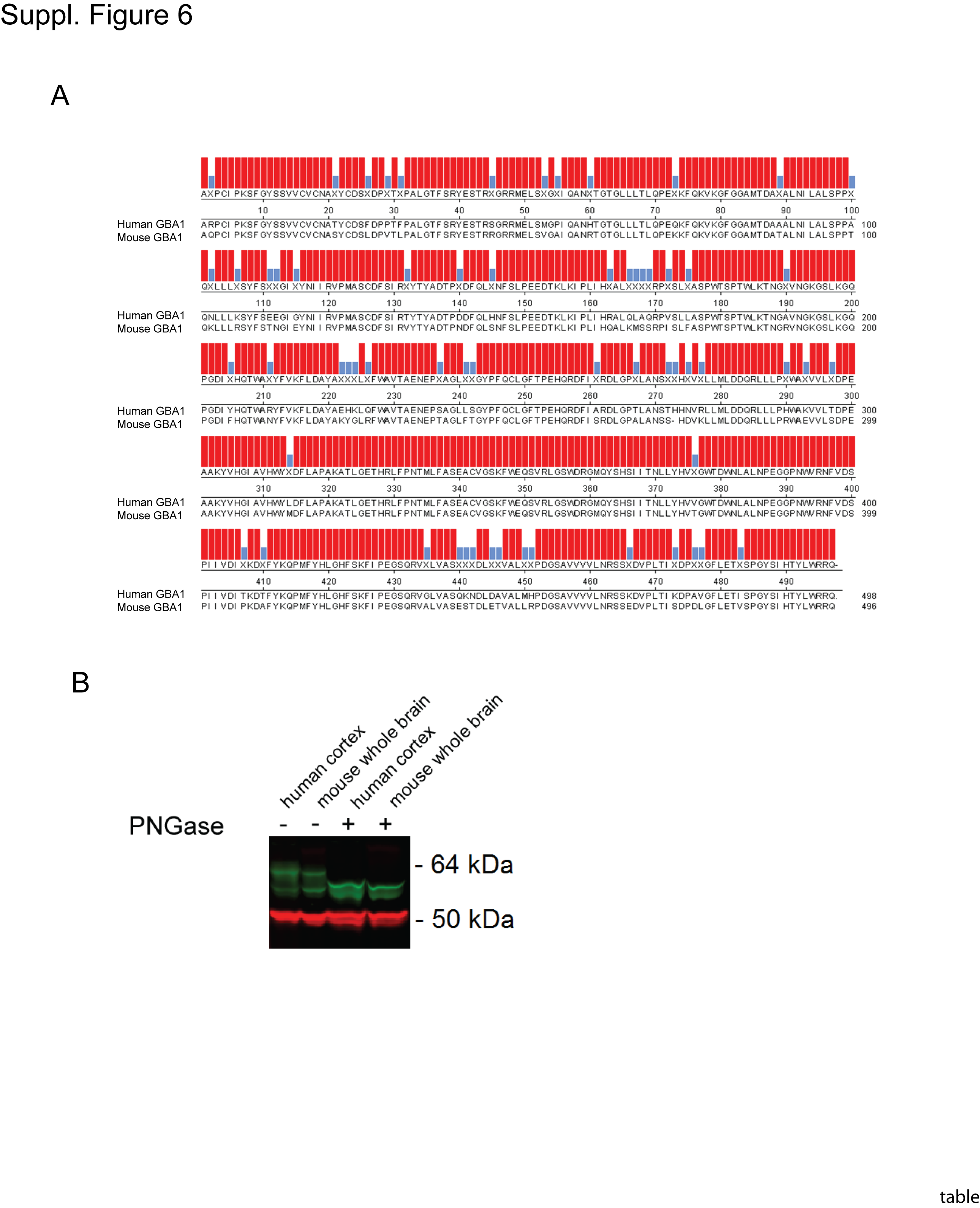

Supplement: S6 Fig — A) Sequence alignment of human and mouse GBA1 proteins. Positions of conservation are indicated by red bars and numbering corresponds to human GBA1. Overall, sequence identity is ~87%. B) Glucocerebrosidase from human and mouse brain migrates at a similar molecular weight on SDS-PAGE/western blot, suggesting a similar extent of glycosylation. Green–GBA1, red–actin. (TIF) [file pone.0119141.s006.tif]

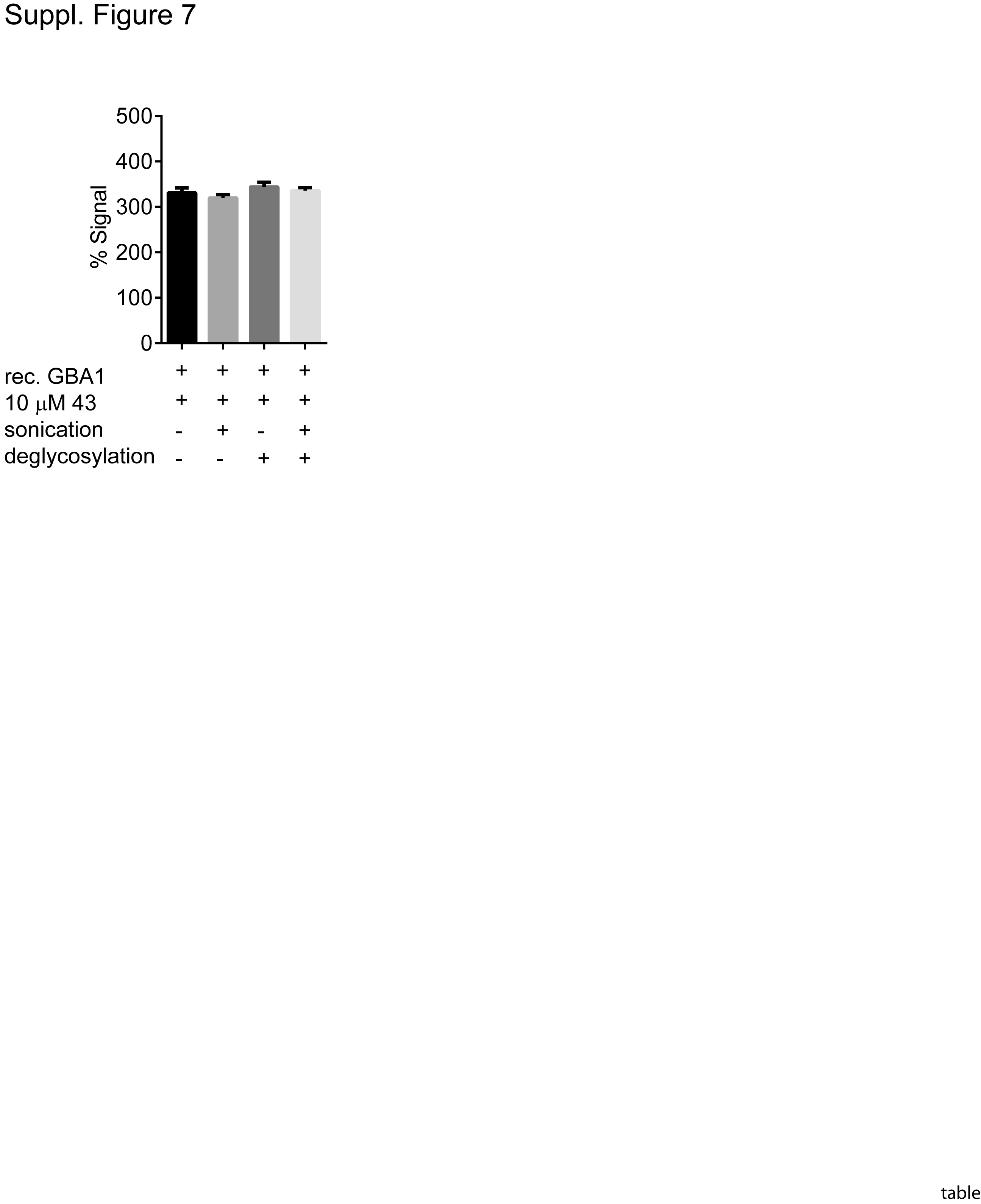

Supplement: S7 Fig — 10 μM compound 43 was used, recombinant GBA1 was sonicated prior to performing the 4MUG enzyme activity assay. (TIF) [file pone.0119141.s007.tif]
